# Linseed oil supplementation affects fatty acid desaturase 2, peroxisome proliferator activated receptor gamma, and insulin-like growth factor 1 gene expression in turkeys (*Meleagris gallopavo*)

**DOI:** 10.5713/ajas.20.0030

**Published:** 2020-06-23

**Authors:** Klaudia Szalai, Károly Tempfli, Eszter Zsédely, Erika Lakatos, András Gáspárdy, Ágnes Bali Papp

**Affiliations:** 1 Department of Animal Science, Faculty of Agricultural and Food Sciences, Széchenyi István University, 9200 Mosonmagyaróvár, Hungary; 2 Department of Food Science, Faculty of Agricultural and Food Sciences, Széchenyi István University, 9200 Mosonmagyaróvár, Hungary; 3 Department of Animal Breeding and Genetics, University of Veterinary Medicine, 1078 Budapest, Hungary

**Keywords:** Fatty Acid Desaturase 2 (*FADS2*), Gene Expression, Insulin-like Growth Factor 1 (*IGF1*), *Meleagris gallopavo*, Peroxisome Proliferator Activated Receptor Gamma (*PPARγ*)

## Abstract

**Objective:**

Effects of linseed oil (LO) supplementation on the fat content and fatty acid profile of breast meat, and the expression of three genes in the liver, breast muscle and fat tissues of commercial 154-day-old hybrid male turkeys were investigated.

**Methods:**

The animals in the control group were fed a commercially available feed and received no LO supplementation (n = 70), whereas animals in the LO group (n = 70) were fed the same basic diet supplemented with LO (day 15 to 21, 0.5%; day 22 to 112, 1%). The effect of dietary LO supplementation on fatty acid composition of breast muscle was examined by gas chromatography, and the expression of fatty acid desaturase 2 (*FADS2*), peroxisome proliferator activated receptor gamma (*PPARγ*), and insulin-like growth factor 1 (*IGF1*) genes was analysed by means of quantitative reverse transcription polymerase chain reaction.

**Results:**

The LO supplementation affected the fatty acid composition of breast muscle. Hepatic *FADS2* levels were considerably lower (p<0.001), while adipose tissue expression was higher (p<0.05) in the control compared to the LO group. The *PPARγ* expression was lower (p<0.05), whereas *IGF1* was higher (p<0.05) in the fat of control animals. There were no significant (p>0.05) differences in *FADS2*, *PPARγ*, and *IGF1* gene expressions of breast muscle; however, omega-6/omega-3 ratio of breast muscle substantially decreased (p<0.001) in the LO group compared to control.

**Conclusion:**

Fatty acid composition of breast meat was positively influenced by LO supplementation without deterioration of fattening parameters. Remarkably, increased *FADS2* expression in the liver of LO supplemented animals was associated with a significantly decreased omega-6/omega-3 ratio, providing a potentially healthier meat product for human consumption. Increased *PPARγ* expression in fat tissue of the LO group was not associated with fat content of muscle, whereas a decreased *IGF1* expression in fat tissue was associated with a trend of decreasing fat content in muscle of the experimental LO group.

## INTRODUCTION

The utilization of long chain polyunsaturated fatty acids (LC-PUFAs), especially the omega-3 type, has health-promoting effects in conditions such as cardiovascular disease, neurological disorders, diabetes, arthritis, inflammation, autoimmune disorders and cancer, as well as the improvement of brain and visual development [1]. Dietary ingestion remains the main and major source of omega-3 PUFAs since the human body is unable to produce it in adequate quantities. There are two common strategies to overcome the problem of low LC-PUFA intake: a pharmacological approach, and the enrichment of food with LC-PUFAs. Linoleic acid (LA; 18:2, n-6) and α-linolenic acid (ALA; 18:3, n-3) are two dietary precursors for omega-6 and omega-3 LC-PUFAs. Linseed oil (LO) is rich in ALA (which makes up about 50% of its total fatty acid content), while it contains less LA than most of the other, often used plant oils [1].

Vertebrates are unable to synthesize the essential fatty acid (EFA), LA, and ALA from acetyl-CoA *de novo*, but can convert EFAs supplied by the diet into more unsaturated fatty acids with a longer carbon chain [2]. Animals can synthesize eicosapentaenoic acid (EPA), docosapentaenoic acid, and docosahexaenoic acid (DHA) from ALA, and arachidonic acid (ARA) from LA. The liver plays the main role in lipid metabolism, which involves the synthesis and modification of fatty acids by way of desaturation, elongation, and oxidation processes [1]. Several studies have reported that it is possible to enrich poultry products (meat and egg) via omega-3 PUFA supplementation of animal feed [3,4].

The effect of LO supplementation on the expression of genes involved in the PUFA metabolism is not clear. Whether the increase in omega-3 PUFAs in turkey muscle, fat and liver following LO supplementation of the feed is related to differential expression of fatty acid desaturase 2 (*FADS2*), peroxisome proliferator activated receptor gamma (*PPARγ*) and insulin-like growth factor 1 (*IGF1*) genes is not known. The objective of this study was to investigate the expression of *FADS2*, *PPARγ*, and *IGF1* in muscle, fat and liver tissues from commercial male turkeys (*Meleagris gallopavo*) by means of quantitative reverse transcription polymerase chain reaction (RT-qPCR), and that whether the expressions of these genes are in line with the fat composition of breast muscle.

## MATERIALS AND METHODS

### Animal care

The experimental procedure was approved by the Institutional Animal Care and Use Committee at Széchenyi István University (MÁB/2018/002).

### Animals and diet

The animals used in this study were kept under identical housing conditions at the experimental farm of Széchenyi István University, Faculty of Agricultural and Food Sciences, located in Mosonmagyaróvár, Hungary. A total of 140 one-day-old Hybrid Converter male turkeys were equally and randomly divided into two feeding groups (control and LO) with two replicates of 35 birds in each. Feeding was done in groups. The animals in the control group were fed a commercially available feed and received no LO supplementation, whereas animals in the LO group were fed the same basic diet supplemented with LO between day 15 to 21 (with 0.5% LO) and day 22 to 112 (with 1% LO). Starter-1 and −2 feedstuffs were in crumble form, whereas grower and finisher feeds were supplied in granulated form. The LO supplementation was terminated in the finisher phases (42 days prior to slaughter). The reason behind this was that we found undesirable side effects (often described as “fishy taste” by consumers) in our previous experiments when LO addition was continued until slaughter (unpublished). By this earlier cessation of supplementation, we aimed to achieve the positive effects of LO without considerably deteriorating flavour. The LO was sprayed onto the feed with continuous mixing to produce a homogeneous mixture. Main details of the diet are presented in Table 1. Live weight, average weight gain, feed intake and feed conversion were recorded at every dietary change throughout the experiment. According to the group mean (average live weight at 148 day) 12-12 birds were selected from both the control and the LO groups for further investigation and sampling for gene expression studies.

### Determination of chemical composition

The determination of chemical composition was performed using the following methods: moisture content: MSZ ISO 1442:2000; protein content: MSZ EN ISO 5983-2:2009; total fat content: MSZ ISO 1443-2002; ash content: MSZ ISO 936: 2000; fatty acid composition: MSZ/EN ISO-12966-2 (determination of fatty acid methyl esters by gas chromatography).

### Tissue sample collection and RNA extraction

For RNA extraction and gene expression analysis, pectoralis major muscle of breast, abdominal fat, and liver (right lobe) samples were collected from a total of 24 turkeys (12-12 from each treatment group) in DNase and RNase free 1.5 mL Eppendorf tubes filled with RNAlater (Qiagen, Venlo, Netherlands) within 15 minutes after slaughter for RNA extraction and gene expression analysis. Samples were then stored at room temperature until processing. Total RNA was extracted from 300 mg of breast muscle, adipose and liver tissue samples using TRIzol Reagent (Thermo Fisher Scientific, Waltham, MA, USA) according to the manufacturer’s protocol. The RNA concentration was assessed by measuring the absorbance at 260 nm using NanoDrop2000 spectrophotometer (Thermo Fisher Scientific, USA). Integrity of isolated RNA was verified by agarose gel electrophoresis and the presence of visible rRNA bands was regarded as a prerequisite for further sample processing. To eliminate potential DNA contamination, extracted total RNA was pre-treated with RQ1 RNase-free DNase (Promega, Madison, WI, USA) following the manufacturer’s instructions.

### Reverse transcription and quantitative polymerase chain reaction

The cDNA was prepared from 1 μg RNA in a 20 μL reaction volume using the iScript cDNA Synthesis kit (Bio-Rad Laboratories, Hercules, CA, USA). The cDNA synthesis was run under the following conditions: 25°C for 5 min, 37°C for 60 min, and 70°C for 5 min. The qPCR was performed on a CFX96 Real-Time PCR Detection System (Bio-Rad Laboratories, USA) in a total volume of 25 μL reaction mixtures containing 1 μL (100 ng) of diluted cDNA, 12.5 μL Maxima SYBR Green qPCR Master Mix (Thermo Fisher Scientific, USA), 1-1 μL of appropriate oligonucleotide primers (0.4 μM; Table 2), and nuclease free water (up to 25 μL final volume). Primers were designed using the Primer3 software based on available sequences (Table 2). The qPCR amplifications were carried out under the following conditions: 10 min at 95°C, followed by 40 cycles of 15 s at 95°C, and 1 min at 60°C. A melting curve analysis (from 65°C to 95°C with 0.5°C increments) for the PCR product was used for each gene to confirm specific amplification of the analysed locus. The expression of the target genes was normalized using glyceraldehyde 3-phosphate dehydrogenase (*GAPDH*) and beta-actin (*ACTB*) as reference genes. The relative gene expression level was calculated according to the comparative threshold cycle method (2^−ΔΔCt^). Each sample was run in triplicates, and each reaction contained no template controls for the genes of interest as well as the reference genes. No-template controls were accepted as negatives with threshold cycles over 35. Reaction efficiency for each run was determined by the application of 10-fold diluted standard samples [5].

### Statistical analysis

Statistical difference between means of live weight at the end of each period, the chemical composition and the fatty acid profile of breast muscle was evaluated by analysis of variance using SPSS Statistics v20.0 (IBM Corp., Armonk, NY, USA). The expression of genes of interest was analysed by means of the 2^−ΔΔCt^ method normalized to two reference genes, namely *GAPDH* and *ACTB.* Within tissue gene expression values distributed normally as tested by the Shapiro–Wilk test in SPSS Statistics v20.0. Statistical analysis was performed on the 2^−ΔΔCt^ values by means of the independent samples t-test in SPSS Statistics v20.0, as well.

## RESULTS

Live weight, average daily weight gain, feed composition, feed intake and feed conversion during the experiment are shown in Table 3. There were no significant (p<0.05) differences between the control and the experimental groups regarding live weights measured at the end of each feeding period.

**Figure 1:**
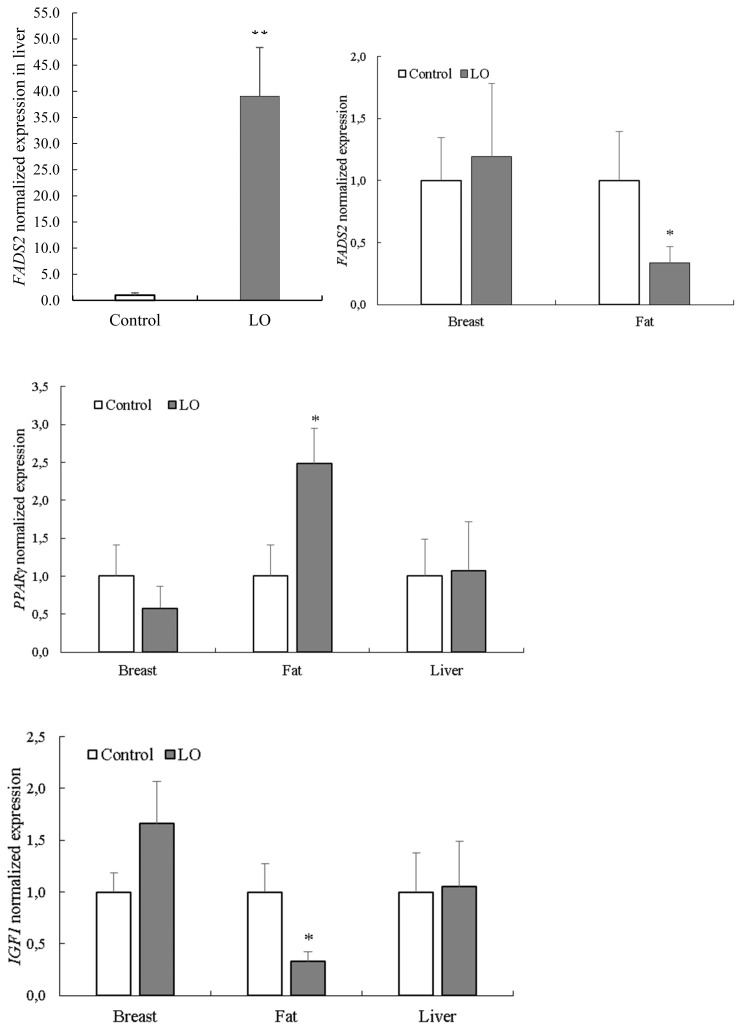
Normalized *FADS2*, *PPARγ*, and *IGF1* gene expression in breast, fat, and liver tissues in male turkeys without or with dietary LO supplementation. Each bar represents the mean±standard error of the mean of groups. LO, linseed oil; *FADS2*, fatty acid desaturase 2; *PPARγ*, peroxisome proliferator activated receptor gamma; *IGF1*, insulin-like growth factor 1. * Indicates significant (p<0.05) differences between treatment groups. ** Indicates significant (p<0.001) difference between treatment groups.

Effects of LO on the chemical composition and fatty acid profile of breast muscle are presented in Table 4. The LO supplementation did not affect the chemical composition (dry matter, protein, ash, fat) of breast muscle, whereas the fatty acid composition of breast muscle was altered. Mainly the fatty acids belonging to the omega-3 group were influenced by the LO supplementation (p<0.01), as observed with ALA (p<0.05), as well. The LO supplementation had no effect on omega-6 fatty acids, including linolenic acid. The ratio of monounsaturated fatty acids and saturated fatty acids were not significantly (p>0.05) influenced by LO supplementation, whereas omega-6/omega-3 ratio was changed (p<0.001) substantially (Table 4). The *FADS2*, *PPARγ*, and *IGF1* expression levels in different tissues are shown in Figure 1. The expression of hepatic *FADS2* was considerably higher (p< 0.001) in birds fed a LO supplemented diet compared to control animals. Conversely, the expression of *FADS2* in fat tissue was significantly higher (p<0.05) in the control group compared to LO-supplemented animals. The mRNA levels of *PPARγ* were higher (p<0.05) in the adipose tissue of birds fed LO supplement. The mRNA levels of *IGF1* were lower (p<0.05) in birds fed a LO supplement. In breast muscle, there were no significant differences between the two groups. In the present experiment LO supplementation slightly reduced the feed intake, while the average daily weight gain means were similar in each feeding periods, which can be explained by increased energy concentration of experimental feed due to LO supplementation. Increasing of energy content by LO supplementation positively influenced the feed conversion during the experiment.

## DISCUSSION

The chemical composition (dry matter, protein, fat, and ash %) of the breast muscle did not change; however, fatty acid composition of breast muscle was affected by LO supplementation. In accordance with our results, other studies [1,3, 6–8] showed that omega-3 content of breast muscle increased and omega-6/omega-3 ratio of breast muscle decreased using linseed supplementation.

The effect of LO supplementation on the expression of genes involved in the PUFA metabolism is not clear [3]. The delta-6 fatty acid desaturase enzyme encoded by *FADS2* gene takes part in the biosynthesis of PUFAs. Delta-6 desaturase puts double bonds in the fatty acids 18:3, n-3 (ALA), 24:5, n-3 (tetracosapentaenoic acid), 18:2, n-6 (LA), and 24:4, n-6 (tetracosatetraenoic acid). The enzyme is regulated by dietary and hormonal factors in mammals [3], as well as by genotype [9]. Research related to the *FADS2* gene in poultry is limited; it is currently not known whether *FADS2* plays a role in poultry growth and development [10]. Desaturase activity is low in non-hepatic tissues [11], and the liver is regarded to be the main site of ARA, EPA, and DHA production for peripheral tissue utilization [12]. Desaturase gene and protein expression and enzymatic activity are primarily influenced by the diet, but age, sex and genetic variations are also influential [4]. The nutritional regulation of *FADS2* has been reported in mammals and chickens. Dinh et al [13] found that in rats an ALA-deficient diet did not affect hepatic *FADS2* activity. Another study concluded that feeding rats with increased ALA diet also did not influence the hepatic expression of desaturase [14]. In contrast, Igarashi et al [15] reported that feeding an ALA deficient diet upregulated the expression and activity of *FADS2* in the liver. In accordance with the present study, Mirshekar et al [6] found an increase in hepatic *FADS2* expression in chickens fed an experimental LO diet. Furthermore, Geay et al [16] observed a significant (p<0.05) increase in hepatic *FADS2* expression in sea bass fed an ALA rich diet. Although the reason for the controversy in these perceptions is unclear, the differences might be due to alterations in the omega-6/omega-3 PUFA ratio, experimental duration or species studied [1,17]. The absolute amount of ALA and LA intake is crucial in regulating the expression and activity of enzymes involved in PUFA conversion. In rodents fed DHA-enriched diets, Nakamura et al [2] observed that a high concentration of long chain PUFAs suppressed the expression and/or activity of fatty acid enzymes. Jing et al [1] also showed that the expression of desaturase and elongase genes in chicken liver were upregulated when the LA:ALA ratio in the diet was reduced. Boschetti et al [9] found that fast growing chickens exhibited lower *FADS2* expression than the slower-growing animals. However, available quantitative data on the expression of lipid-related enzymes are scarce in turkey.

The *PPARγ* is a member of the nuclear receptor family of ligand-activated transcription factors. *PPARγ* is the primary regulator of adipogenesis and lipogenesis in mammals and birds, and plays important roles in the development of obesity, the pathology of diabetes, atherosclerosis, and cancer [18]. The *PPARγ* protein forms obligate heterodimers with the retinoid X receptor to regulate the transcription of genes involved in glucose and lipid metabolism, and adipocyte differentiation [19]. The *PPARγ* gene is a candidate gene for abdominal fat deposition [20] and may also be responsible for intramuscular fat accumulation in chicken [21]. The *PPARγ* gene encodes peroxisome proliferator-activated receptor gamma, an enzyme that participates in adipogenesis and lipogenesis in mammals and birds and plays important roles in the development of obesity [18]. Hyperexpression of *PPARγ* is associated with obesity in humans [22], and *PPARγ* expression is also correlated with fat deposition in broilers [23]. Larkina et al [20] found that *PPARγ* mRNA levels were higher in the liver of fat broilers compared to lean ones. There was a strong correlation between *PPARγ* expression and abdominal fat content, as well as fat weight; however, there was no difference in *PPARγ* expression in adipose tissue between fat and lean groups. Fu et al [24] found that *PPARγ* expression in abdominal fat was significantly higher than that in breast and thigh muscle at all examined stages (day of hatching; 4, 8, 14 and 20 weeks of age) in chickens. It was also reported that *PPARγ* could be responsible for intramuscular fat deposition in chickens [21]. In our study, *PPARγ* mRNA level in the adipose tissue of LO group significantly (p<0.05) exceeded *PPARγ* mRNA level of control group, without any differences of other investigated tissues (i.e. liver and breast).

Insulin-like growth factors (IGF) have been well-studied and the *IGF1* gene is known to play a pivotal role in chicken muscle development. In birds, *IGF1* is essential for normal growth and development. The IGF1 might be derived from increased synthesis in the liver under the effect of growth hormone, and mostly may be of local origin, and has autocrine or paracrine effects. It is also known that *IGF1* has an important role in carbohydrate, fat, and protein metabolism in several tissues, e.g. muscle, fat, and liver. In skeletal muscle cells *IGF1* stimulates protein synthesis and glucose uptake. Diets with elevated omega-3 PUFA content might affect *IGF1* expression since *IGF1* mRNA level in liver and muscle greatly depends on nutritional status [25,26]. In birds, the liver is not the only source of *IGF1*, as it is synthesized in several other tissues, including brain, eye, lung, pancreas, and muscles [27]. The *IGF1* gene plays an important role in the metabolism of carbohydrates, fats and proteins in adipose tissue, skeletal muscle, and liver [28]. Consistent with the present study, Wei et al [29] observed that pigs fed a linseed-enriched diet for 30 and 60 day had a higher expression of *IGF1* in their skeletal muscle compared to those with no linseed supplementation, suggesting that feeding a linseed-enriched diet may improve the growth of skeletal muscle. Feeding pigs with a linseed-enriched diet might also increase the insulin-induced protein synthesis in skeletal muscle. Fasting led to a reduction in *IGF1* mRNA levels in the muscle [26] and in liver [25] of broiler chickens. Saprõkina et al [7] showed that the effect of absorbed PUFA is quantitatively related to the *IGF1* gene expression level in several tissues in chickens; however, the exact mechanism through which PUFA affects *IGF1* gene expression is still not known. Chickens with higher growth rates showed higher *IGF1* protein and mRNA levels in their liver [30]. The *IGF1* concentration in blood serum and its relative mRNA concentration in leukocytes tended to be lower in quails fed linseed-modified diets, but the changes were not significant [8].

In our study, in breast muscle of LO supplemented animals increased PUFA, omega-3 content, and decreased omega-6/omega-3 ratio were detected compared to the control animals, despite of the fact, that in breast muscle there were no significant (p>0.05) differences between the *FADS2*, *PPARγ*, and *IGF1* expression levels of the LO and the control group. The present study concluded that dietary LO supplementation affects the expression of *FADS2*, *PPARγ*, and *IGF1* genes related to fat metabolism and growth in breast muscle, adipose and liver tissues in hybrid male turkeys. Differential expression of the analysed genes can contribute to the growth and elevated PUFA content in the animals. Further investigations with modified experimental design (e.g. increased and extended LO supplementation) are needed to address inconsistencies between differential gene expression and changes in breast muscle lipid profile and fat content. From a human nutritional point of view, LO supplementation of turkey feedstuffs results in beneficial effects.

## Figures and Tables

**Table 1:** Nutrient content of different diets

Items	Starter-1 1–14 d		Starter-2 15–21 d		Grower-1 22–84 d		Grower-2 85–112 d		Finisher-1 113–133 d		Finisher-2 134–154 d	
					
	Control	LO	Control	LO 0.5%	Control	LO 1%	Control	LO 1%	Control	LO	Control	LO
DM (%)	90.9	89.1	91.5	93.5	91.3	93.3	92.4	93.3	92.2	93.3	91.1	91.6
Crude protein (g/kg DM)	286.0	284.4	290.9	293.0	244.2	241.2	221.9	221.9	194.1	192.9	185.5	182.3
Crude fat (g/kg DM)	46.2	52.3	65.9	57.8	72.3	84.7	69.3	79.3	68.3	81.5	86.7	98.3
Crude ash (g/kg DM)	73.7	-	77.3	77.0	65.7	65.4	57.4	56.8	43.4	50.4	46.1	48.0
AMEn (MJ/kg DM)	12.4	12.4	13.4	12.8	12.6	13.0	12.9	13.1	13.1	13.4	14.2	13.7
SFA (% ot total FA)	15.2	15.5	13.6	13.1	12.8	12.5	16.1	16.2	16.5	15.5	37.9	13.2
MUFA (% ot total FA)	23.4	22.9	24.5	24.4	25.3	25.0	26.5	26.4	27.1	26.6	40.8	28.8
PUFA (% of total FA)	61.4	61.4	61.8	62.5	61.9	62.5	57.4	57.3	56.5	58.0	21.3	57.9
Omega-6 (% of total FA)	55.7	48.3	58.0	51.9	59.2	54.2	52.6	50.2	52.0	48.9	20.3	53.4
Omega-3 (% of total FA)	5.8	13.1	3.8	10.6	2.7	8.3	4.8	7.1	4.4	9.0	1.9	4.5
Omega-6/Omega-3	9.6	3.7	15.3	4.9	22.0	6.5	10.9	7.0	11.7	5.4	11.0	11.9
Ca (%)	1.4	na	1.3	na	1.2	na	1.1	na	0.9	na	0.8	na
P (%)	0.9	na	0.9	na	0.8	na	0.7	na	0.6	na	0.6	na
P (% utilized)	0.6	na	0.6	na	0.5	na	0.4	na	0.3	na	0.3	na
Lysine (%)	1.8	na	1.7	na	1.5	na	1.3	na	1.1	na	0.9	na
Methionine (%)	0.6	na	0.6	na	0.5	na	0.4	na	0.4	na	0.3	na
Methionine+cystine (%)	1.1	na	1.0	na	0.9	na	0.8	na	0.7	na	0.6	na
Threonine (%)	1.1	na	1.0	na	0.9	na	0.8	na	0.7	na	0.6	na

LO, linseed oil; DM, dry matter; AMEn, apparent metabolizable energy; SFA, saturated fatty acid; FA, fatty acid; MUFA, monounsaturated fatty acid; PUFA, polyunsaturated fatty acid; na, not analysed separately in the feed of the LO group.

**Table 2:** Primer sequence, amplicon size, and qPCR efficiency

Gene	Reference sequence	Primer sequence (forward/reverse)	Amplicon size (bp)	Efficiency (%)
*ACTB*	AY942620.1	CTGGCACCTAGCACAATGAA/GCTGGAAGGTGGACAGAGAG	103	91.2±6.0
*GAPDH*	NM_001303179.1	GCAGATGCAGGTGCTGAGTA/CACAAACATGGGAGCATCAG	135	90.0±6.7
*FADS2*	XM_010711278.2	GTTCACCGGACACCTGAACT/TGGACTCCATACTTGGCACA	117	93.8±4.2
*PPARγ*	XM_010718432.2	TTGCCAAAGTGCAATCAAAA/TGAAATCCAGAGGCCTTGTC	147	95.2±5.5
*IGF1*	NM_001303149.1	CGCTTACACCACAAGGGAAT/CACGTACAGAGCGTGCAGAT	115	93.7±6.2

qPCR, quantitative polymerase chain reaction; *ACTB*, beta-actin; *GAPDH*, glyceraldehyde 3-phosphate dehydrogenase; *FADS2*, fatty acid desaturase 2; *PPARγ*, peroxisome proliferator activated receptor gamma; *IGF1*, insulin-like growth factor 1.

**Table 3:** Live weight, weight gain, feed intake and feed conversion in each period

Items	Live weight		Average weight gain (g/bird/d)		Average feed intake (g/bird/d)		Average feed conversion (kg/kg)	
			
	Control (n = 70)	LO group (n = 70)	Control	LO group	Control	LO group	Control	LO group
Starter-1	1.23±0.15	1.19±0.18	41.32	39.86	53.72	51.87	1.30	1.30
Starter-2	3.43±0.57	3.35±0.42	136.76	132.48	203.22	196.76	1.49	1.49
Grower-1	5.64±0.62	5.60±0.64	176.40	179.35	324.88	319.28	1.84	1.78
Grower-2	9.72±1.04	9.78±0.97	194.56	196.42	460.81	454.72	2.37	2.32
Finisher-1	12.62±1.38	12.69±1.10	202.54	206.79	615.29	603.12	3.04	2.92
Finisher-2	23.69±2.81	23.47±1.77	173.45	187.09	725.77	713.37	4.19	3.82

LO, linseed oil.

**Table 4:** Effect of linseed oil on the chemical composition and the fatty acid profile of breast muscle (% of total fatty acids)

Items	Control	LO
Dry matter (%)	25.8±1.0	26.2±0.6
Protein (%)	22.8±0.3	23.4±0.9
Ash (%)	1.2±0.0	1.1±0.1
Fat content (%)	1.6±0.6	1.5±0.4
SFA (%)	28.73±1.7	26.41±2.03
Myristic acid (C14:0)	0.22±0.10	0.20±0.03
Palmitic acid (C16:0)	20.26±1.80	16.26±2.05**
Stearic acid (C18:0)	7.69±1.40	9.43±1.66
MUFA (%)	28.99±2.24	27.09±2.60
Elaidic acid+oleic acid (C18:ln9)	25.37±1.15	24.56±2.10
Vaccenic acid (C18:ln7)	1.31±0.17	1.32±0.13
PUFA (%)	42.29±2.88	46.51±2.72**
Linolenic acid (C18:2)	37.60±2.56	38.00±2.32
α-Linoleic acid (C18:3n3)	1.64±0.17	2.61±0.74*
Arachidonic acid (C20:4n6)	2.34±0.86	4.23±2.31
EPA (C20:5n3)	0.04±0.01	0.09±0.04*
DPA (C22:5n3)	0.20±0.08	0.67±0.32*
DHA (C22:6n3)	0.09±0.04	0.12±0.05
Omega-6 (%)	40.31±2.73	42.76±2.41
Omega-3 (%)	1.98±0.19	3.74±0.68**
Omega-6/Omega-3	20.41±1.01	11.79±2.54***

LO, linseed oil; SFA, saturated fatty acid; MUFA, monounsaturated fatty acid; PUFA, polyunsaturated fatty acid; EPA, eicozapentaenoic acid; DPA, docosapentaenoic acid; DHA, docosahexaenoic acid; ns, not significant (p>0.05);

* p<0.05;

** p<0.01;

*** p<0.001, the LO group is significantly different compared to control.
